# Operating Cost and Treatment of Boron from Aqueous Solutions by Electrocoagulation in Low Concentration

**DOI:** 10.1002/gch2.201800011

**Published:** 2018-04-20

**Authors:** Fatma Deniz, Ceyhun Akarsu

**Affiliations:** ^1^ Department of Environmental Engineering Engineering Faculty Mersin University 33343 Mersin Turkey

**Keywords:** aqueous solutions, boron removal, electrocoagulation process, iron electrodes, optimization

## Abstract

The objective of this study is to determine the optimum parameters of electrocoagulation process in treatment of boron in low concentrations. Especially, studies on electrode optimization in low boron concentrated waters are insufficient. Therefore, the effect of electrode combination (Al–Al, Al–Fe, Al–SS, Fe–Al, Fe–Fe, and Fe–SS), pH (5–9), current density (8–24 mA cm^−2^), distance (1–3 cm), and electrolysis time (10–90 min) on treatment of boron containing wastewater is studied to obtain maximum removal efficiency. The maximum removal efficiency of boron is obtained as 95.6%. Operation conditions for maximum removal are the electrode combination of Fe–Al, current density of 16 mA cm^−2^, pH 7.0, concentration of 30 mg L^−1^ and the reaction time of 70 min. Operating cost of the electrocoagulation process is calculated as 2.35 $ m^−3^. This study indicates that the electrocoagulation process can be successfully applied in order to treat boron‐polluted wastewaters at low initial concentrations.

## 1 Introduction

Boron is the 51st element that is common in the earth's crust and it is almost never free form in nature.^1^ It is known that there are about 230 kinds of boron minerals in the soil. The different properties exhibited by the various metal or nonmetal compounds make it possible to use many of the boron compounds in the industry.^2^ Boron is also found in surface water at around 4.5 mg L^−1^.^3^ The long‐term use of water containing boron can cause toxic effects for plants, animals, and humans.^4^, ^5^, ^6^ Because of these reasons, the boron concentration in drinking water has been set in as 2.4 and 1 mg L^−1^ by WHO^7^ and by EU,^8^ respectively.

Boron compounds are widely used in the manufacture of ceramics, glass, detergents, high quality steel, catalysts, cosmetics, and flame retardants.^9^, ^10^ Hence, these industries produce boron‐containing wastewater. The amount of water containing boron, and boron concentration of wastewater varies from industries.^11^ Plants are particularly sensitive to boron concentration (<0.75 ppm). Treatment of wastewaters is an important issue to maintain environment health.^12^ For reducing boron concentration in wastewater several techniques have been studied such as ion exchange,^13^ adsorption,^14^ chemical precipitation,^15^ reverse osmosis,^16^ bio electrochemical reactor,^17^ and MFC.^18^

Coagulation is a common method of the treatment of heavily contaminated wastewater, e.g., industrial wastewater.^19^ The process is based on the use of FeCl_3_, Al_2_(SO_4_)_3_ as coagulant reagents.^20^ However, its main disadvantage is the formation of large amounts of solid wastes and mud after coagulation as well as the removed but not degraded contaminants.^21^ Electrocoagulation process (EC) is based on producing of coagulants from the appropriate anode such as iron and/or aluminum which is oxidized due to the applied current. Electrolysis gases as hydrogen gas evolves at the cathode and helps remove pollutants by flotation.^22^, ^23^

Reactions that occur at the anode and cathode electrodes for aluminum and iron electrodes in an EC cell are described in Equations (1)–(7).^3^, ^22^, ^24^

Anode:

(1) $$\mathrm{Al}\to {\mathrm{Al}}^{3+}+3e^{-}$$

Cathode:

(2) $$2H_{2}O+2e^{-}\to H_{2}+2{\mathrm{OH}}^{-}$$

In the solution:

(3) $${\mathrm{Al}}^{3+}+3{\mathrm{OH}}^{-}\to \mathrm{Al}{\left(\mathrm{OH}\right)}_{3}$$

Anode:

(4) $$4{\mathrm{Fe}}_{\left(s\right)}\to 4{\mathrm{Fe}}^{\mathrm{2+}}{}_{\left(\mathrm{aq}\right)}+8e^{-}$$

(5) $$4{\mathrm{Fe}}^{\mathrm{2+}}{}_{\left(\mathrm{aq}\right)}+10H_{2}O_{\left(I\right)}+O_{2}\to 4\mathrm{Fe}{\left(\mathrm{OH}\right)}_{3(s)}+8H^{+}{}_{\left(\mathrm{aq}\right)}$$

Cathode:

(6) $$8H^{+}{}_{\left(\mathrm{aq}\right)}+8e^{-}\to {\mathrm{4H}}_{2(g)}$$

Overall:

(7) $$4{\mathrm{Fe}}_{\left(s\right)}+10H_{2}O_{\left(I\right)}+O_{2}\to 4\mathrm{Fe}{\left(\mathrm{OH}\right)}_{3(s)}+4H_{2(g)}$$

Electrocoagulation process (EC) has been used for treatment of many industrial effluents due to its advantages such as low operating costs, easy operation, simple equipment, ecofriendly in nature and relatively low amount of sludge generation.^25^, ^26^, ^27^ EC has been successfully used for the removal of oil and grease from biodiesel wastewater, with a removal efficiency as high as 98.42%.^28^ Similar successful results were obtained from treatment of antibiotics,^19^ textile wastewater^29^ phosphate from surface water,^30^ lead,^31^ landfill leachate,^32^ phenolic compounds,^21^ and heavy metals.^33^, ^34^

Although, there are some studies regarding the removal of boron by electrocoagulation process,^10^, ^35^, ^36^, ^37^ its lack of full scale optimization on boron removal in low concentration by electrocoagulation process, especially electrode optimization which causes that makes difference on results and also lack of operating cost calculation. This study aims to investigate removal of boron from aqueous solution by electrocoagulation process at concentrations of less than 50 ppm. Process parameters, such as electrode combination (Al–Al, Al–Fe, Al‐SS, Fe–Al, Fe–Fe, and Fe–SS), pH (5–9), current density (8–24 mA cm^−2^), distance (1–3 cm), initial concentration (10–50 mg L^−1^) and electrolysis time (10–90 min) can significantly affect the performance of EC treatment. These parameters are therefore studied and optimized in this study.

## 2 Results and Discussion

### 2.1 Effect of Electrode Combination

**Figure 1:**
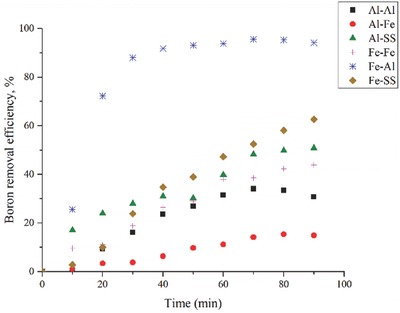
Effect of electrode pair on boron removal versus time (experimental conditions: initial concentration, 30 mg L^−1^; pH 7; electrode distance, 20 mm; current density, 16 mA cm^−2^).

The chemical reactions in the EC process on electrodes are given in Equations (1), (2), (3). The electrode combination is an important factor, which affects the efficiency of the EC process.^38^, ^39^, ^40^ Aluminum, iron, and stainless steel were used in six different combinations to evaluate the optimum electrode pair and the results are given in **Figure** **1**. Boron concentration was reduced to less than 1.35 mg L^−1^ which means 95.6% removal efficiency is succeeded with the usage of Fe–Al electrodes. However, maximum boron removal efficiencies were 34%, 15%, 50%, 44%, and 63% with the usage of Al–Al, Al–Fe, Al–SS, Fe–Fe, and Fe–SS electrodes, respectively. Hence, electrode optimization in electrochemical treatment studies has great importance and should be performed at every study.

Fe–Al electrode combination supplied the highest boric acid removal. This can be explained by oxidation potential of iron (−0.447 V) compared to that for aluminum (−1.662 V),^41^ and also iron electrodes can produce more bubbles than aluminum electrodes.^42^

### 2.2 Effect of pH

**Figure 2:**
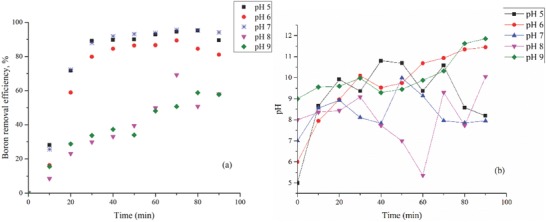
a) Effect of pH on boron removal versus time (experimental conditions: initial concentration, 30 mg L^−1^; electrodes, Fe–Al; electrode distance, 20 mm; current density, 16 mA cm^−2^). b) pH changes in the reactor during pH optimization study.

The results of the effect of initial pH on boron removal are shown in **Figure** **2**a.The pH values of the sample were also measured after treatment and it is shown in Figure 2b.These results are consistent with the results of previous studies.^40^

The pH of the solution increased for all applied potentials after electrocoagulation. For example, the pH values increased from initial pH 7.00 to 8.00 ± 0.3 in electrode optimization. It was observed that there was an independent increase from the electrode. It is probably because of the removal of the boric acid from the solution. However, later in the optimization steps, a significant increase in pH was observed. This result could be explained by the reactions, which take place at the Fe anode and Al cathode.

### 2.3 Effect of Current Density

**Figure 3:**
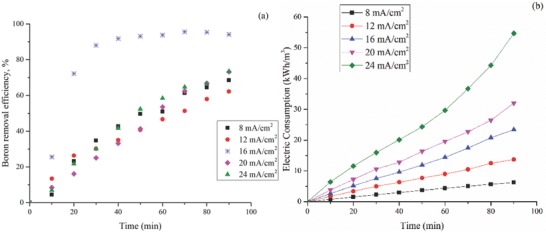
a) Effect of current density on boron removal versus time (experimental conditions: electrodes, Fe–Al; pH 7; electrode distance, 20 mm; concentration, 30 mg L^−1^). b) Energy consumption calculations depending on the voltage changes in the reactor.

The results of the current density studies are shown in **Figure** **3**a. The highest efficiency was obtained as 95.6% with 16 mA cm^2^. It was seen that the efficiency of boron removing increased as the applied current density increased till 16 mA cm^−2^. Further increases of current density (20–24 mA cm^−2^) did not provide better removal efficiency as seen in Figure 3a. Yavuz and Ogutveren^26^ have obtained the same increase and decrease of removal efficiency in current density optimization study with iron electrode. The obtained results for energy consumption were also given in Figure 3b.

### 2.4 Effect of Interelectrode Distance

Dolati et al.^3^ found that the efficiency of the EC process increased with the decrease in the distance between the electrodes. Verma^29^ noticed that the effect of inter electrode distance was related to the conductivity.

**Figure 4:**
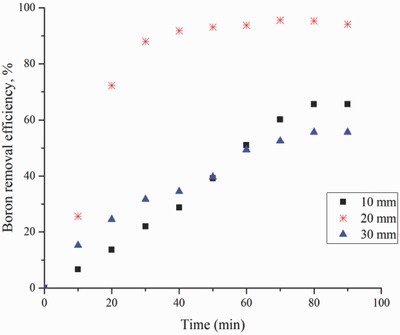
Effect of inter‐electrode distance on boron removal versus time (experimental conditions: electrodes, Fe–Al; pH 7; concentration, 30 mg L^−1^; current density, 16 mA cm^−2^).

The results of the effect of interelectrode distance are shown in **Figure** **4**. Maximum boron removal efficiency of 95.6% was obtained with a distance of 20 mm between the electrode pair. As the distance between the electrodes decreased, the efficiency of the AT process decreased. The same proportional results have been obtained by Ezechi et al.^40^

The voltages applied during the operation were recorded. It was found that while the electrode distances were 3, 2, and 1 cm, the voltage values were 23.4, 15.2, and 11 V, respectively, for the same current density (16 mA cm^−2^).

### 2.5 Effect of Initial Boron Concentration

**Figure 5:**
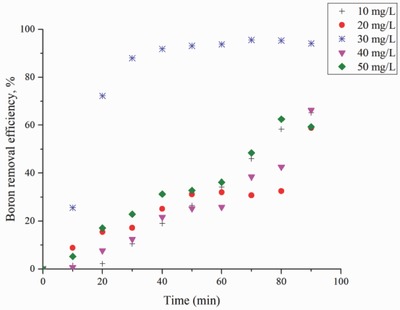
Effect of initial boron concentration on boron removal efficiency versus time (experimental conditions: electrodes, Fe–Al; pH, 7; electrode distance, 20 mm; current density, 16 mA cm^−2^).

**Figure** **5** shows residual boron concentrations for different initial boron concentrations. Residual boron concentrations of 3.49, 8.24, 1.34, 13.50, and 18.74 mg L^−1^ were obtained from initial boron concentrations of 10, 20, 30, 40, and 50 mg L^−1^, respectively. The decrease in boron removal can be explained by the fact that the generated iron ions are not sufficient.

According to the other investigators,^3^, ^43^ EC can be used for treatment of boron‐polluted water and wastewater containing boron at initial concentrations of more than 50 mg L^−1^. However, it is seen that the removal efficiency of EC is higher at the lower concentrations of 50 mg L^−1^ in the right optimization conditions.

### 2.6 Operating Cost of the EC

When practical application of any treatment method is considered, economic analyses should also be carried out.^44^ Details on operating cost calculation are given in the text above. Energy and electrode consumptions for current density of 16 mA cm^−2^ were determined as 17.33 kWh m^−3^ and 1.2 kg m^−3^ Fe–Al electrodes at 70 min, respectively.

(8) $$\begin{array}{c} \mathrm{OC}=17.33\;\mathrm{kWh}\;m^{-3}\times 0.09\;\$\;{\mathrm{kWh}}^{-1}+1.2\;\mathrm{kg}\;m^{-3}\times 0.66\;\$\;{\mathrm{kg}}^{-1}\\ =2.35\;\$\;m^{-3} \end{array}$$

When the current studies on the boron removal are examined, it is seen that there is not any operating cost calculation in the literature. Therefore, it is not possible to compare the cost calculation with similar studies. Nevertheless, to give example, Yavuz et al.^21^ found that the required cost to treat organic containing effluents (COD), using EC cell, at a current density of 50 mA cm^−2^ was about 2.72 $ m^−3^. Electric consumption decrease 40% as operating time decrease from 70 to 40 min with 92% removal efficiency.

### 2.7 Determination of Kinetics of Boron Adsorption

**Figure 6:**
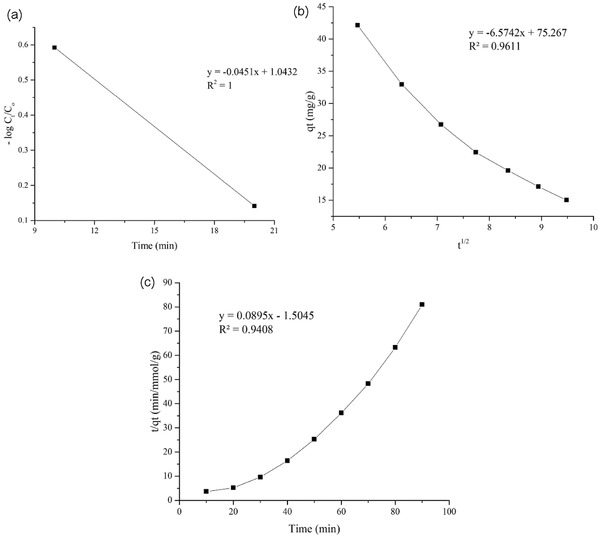
a,b) Effect of the diffusion of the boundary layer; c) boron adsorption in the second steps corresponds to the pseudo‐second‐order model.

Examination of the adsorption kinetics is an important step in understanding the adsorption steps affecting the speed of the adsorption process.^45^ Although, there are four main steps in adsorption process in a solution, the other investigators^46^, ^47^ indicate that the steps 2 and 3 are speed determinations, since steps 1 and 4 will not have an adverse effect on the adsorption rate in good mixing condition. Within a few minutes of the adsorption process, a time versus ‐log *C*_t_/*C*_0_ value is plotted to see if boundary layer diffusion is affected. It can be said that the effect of the diffusion of the boundary layer is so remarkable when linearity of calibration curve was approaching to a straight line (**Figure** **6**a,b).^48^

As seen in Figure 6c, the boron adsorption rate in the reaction time of 10 to 20 min (the step 1 or production of coagulant) is lower than the reaction time of 20 to 90 min (stage 2 or pollutant removal by coagulant). The kinetics of boron adsorption in the second steps corresponds to the pseudo‐second‐order model (*R* ^2^ = 0.94), and they can be used to determine the amount of boron adsorption.

### 2.8 Characterization of Precipitates after EC of Boric Acid

**Figure 7:**
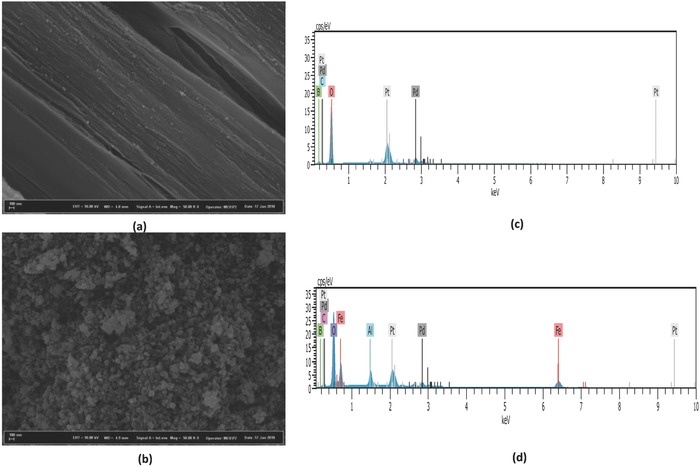
a,b) SEM images of the boric acid and sludge sample after treatment; c,d) EDX analysis of boric acid and sludge sample after treatment.

The surface morphologies of the boric acid (synthetic product) (**Figure** **7**a), and the sample taken from the sludge (Figure 7b) after the treatment were analyzed by FE‐SEM after coating with platinum‐palladium. SEM observation indicates that boric acid has a substantially flat surface with fibrous roughness white sludge has a composed of irregular‐shape crystallites. Energy dispersive X‐ray spectroscopy (EDX) spectra shows that boron compounds constitute 8.52% of total sludge which may causes of low initial concentrations.^43^

## 3 Concluding Remarks

Electrocoagulation experiments with different electrode combinations were conducted to treat boron from aqueous solution in a batch system. Various operating parameters, such as electrode combination, initial pH, current density, electrode distance and initial boron concentration were evaluated to define optimum conditions. The efficiency of this process in boron removal from water at initial concentration of 30 mg L^−1^ under optimal conditions (Fe–Al electrodes, pH 7, electrode distance = 20 mm, reaction time = 70 min, current density = 15 mA cm^−2^) is 95.6%. Increasing boron concentration (40–50 ppm) decreased boron removal efficiency. This increase decreased energy consumption.

## 4 Experimental Section

*Boron‐Containing Aqueous Solution*: Synthetic wastewater prepared with appropriate amount of boric acid (H_3_BO_3_) and dissolved in 1 L distilled water to yield varying boron concentrations of 10, 20, 30, 40, and 50 mg L^−1^. A pH meter (Thermo ‐Orion 3 Star) and a conductivity meter (Hach‐Lange HQ40d) were used to measure the pH and conductivity of the sample respectively.H_2_SO_4_ (0.1–1 N) and NaOH(0.1–1 N) were used to adjust the pH value and NaCl was used to adjust conductivity (2000 ± 50 µs cm^−1^). All chemicals were purchased from Merck Company. Deionized water (Millipore Direct‐Q3UV) was used to prepare the solutions.

**Figure 8:**
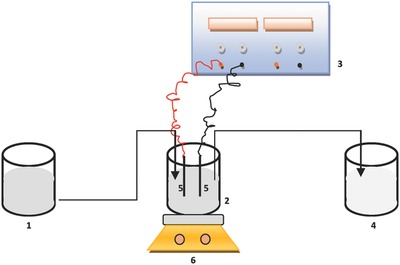
Schematic illustration of EC reactor (1. Storage tank, 2. EC reactor, 3. DC power supply, 4. Treated water storage tank, 5. Anode and cathode, 6. Magnetic stirrer).

*Reactor Setup*: The EC reactor setup scheme is shown in **Figure** **8**.Experiments were carried out in a beaker made of glass material (10.5 cm (radius) × 14.5 cm (deep)) with a certain active capacity (800 mL). Al–Al, Al–Fe, Al–SS, Fe–Al, Fe–Fe, and Fe–SS were used as the anode–cathode electrodes. The surface area of electrodes was 6 cm × 8.5 cm (width × height) and the dimension of electrodes was 1 mm as thickness. The electrodes were connected to a digital DC power supply (AATech ADC‐3303D). After each run of the experiments, the used electrodes were dipped in acid solution for 5 min and rinsed with deionized water and dried for 5 min at 105 °C to remove surface impurities before reuse. Throughout the experiments, the samples were taken at every 10 min and centrifuged (6.000 rpm, 5 min) (Hettich‐ZentrufugenEBA20). To calculate electrode consumption for operating cost, all electrodes were weighed after each run.

Optimization study was carried out for different electrode pairs, pH, current density, distance between electrodes and initial boron concentration.

The electrode combination is an important factor which affects the efficiency of the EC process.^38^, ^39^, ^40^ To determine the optimum electrode pair, Al–Al, Al–Fe, Al–SS, Fe–Al, Fe–Fe, and Fe–SS were used as the anode/cathode electrodes. Other experimental conditions were 30 mg L^−1^ initial concentration, pH 7, 20 mm electrode spacing and 16 mA cm^−2^ current density.

The effect of pH was investigated with the different pH values (5.0–9.0). Electrode pair, current density, electrode distance, and boron concentration were set as Fe–Al, 16 mA cm^−2^, 2 cm, and 30 mg L^−1^, respectively. Residual boron concentrations were analyzed by the carmine method.^49^

Different current density values (8, 12, 16, 20, and 24 mA cm^−2^) were studied to determine the optimum current density value. Electrode pairs were Fe–Al. Other experimental conditions were 30 mg L^−1^ initial concentration, pH 7 and 20 mm electrode distance.

Current density is one another important factor for electrochemical treatment methods because it adjusts bubble production, determines production rate of coagulant and affects the growth of flocs.^50^, ^51^

For optimization of distance between electrodes, 10–20 and 30 mm of electrode distance was studied. Other experimental conditions were Fe–Al electrode pair, 30 mg L^−1^ initial concentration, pH 7 and 16 mA cm^−2^ current density.

To determine optimum initial concentration, different initial concentration of boron (10, 20, 30, 40, and 50 mg L^−1^) was studied. Other experimental conditions were Fe–Al electrode pair, pH 7, 20 mm electrode distance and 16 mA cm^−2^ current density.

To determine efficiency of EC process after each set, residual boron concentrations were analyzed by the carmine method.^49^ The pH values of the samples were also measured after treatment.

Before each run of the experiments, the used electrodes were dipped in acid solution for 5 min, rinsed with deionized water and dried for 5 min at 105 °C to remove impurities from surface. To calculate electrode consumption for operating cost, all electrodes were weighed after each run.

*The Method of Analysis*: Boron concentration was analyzed by the Carmine Method.^33^ Before analyzing, all samples were centrifuged for 5 min in 6000 rpm. Boron removal efficiency was calculated by Equation (9)

(9) $$\mathrm{Boron}\;\mathrm{removal}\;\mathrm{efficiency}\;=\;\left(\frac{B1\;-\;B2}{B1}\right)\;\times \;100$$

where *B*1 is the inlet boron concentration (mg L^−1^) and *B*2 is the boron concentration value at any reaction time of *t* (mg L^−1^).

Field emission scanning electron microscopy (FE‐SEM) images were recorded using a Zeiss/Supra 55 FE‐SEM.

Operating Cost (OC): In this part of the work, the operating cost of the treated boron containing aqueous solution can be calculated by considering 2 major parameters electrode material and amount of energy consumption in the EC process. *C*_energy_ and *C*_electrode_ consumptions are calculated with the following equations^34^

(10) $$C_{\mathrm{energy}}=\frac{U\times i\times t}{\nu }$$

where *C*_energy_ (kWh m^−3^) is electrical energy consumption, *U* is voltage(*V*), *i* is applied current (*A*), *t* is treatment time (h), *v* is the active volume (m^3^).

(11) $$C_{\mathrm{electrode}}=\frac{i\;\times \;t\;\times \;M}{z\;\times \;F\;\times \;v}$$

where *C*_electrode_ is electrode consumption (kg m^−3^) in theoretically, *i* is applied current (A), *t* is treatment time (h), *M* is molecular weight of anode (Fe) (55.85 g mol^−1^), *z* is number of electron involved in the reaction (z_Fe_ = 3), and *F* is the Faraday's constant (96 485 C mol^−1^).

$$\mathrm{OC}=\alpha \;\times \;C_{\mathrm{energy}}+\beta \times C_{\mathrm{electrode}}$$

According to the Turkish market in January 2017, price for electrical energy was 0.09 US $ kWh^−1^ (α), and price for Fe electrode materials was 0.66 US $ kg^−1^ (β).

## Conflict of Interest

The authors declare no conflict of interest.
